# Be in the room where it happens: Building a career at the frontier of new technology

**DOI:** 10.1016/j.hrcr.2026.06.022

**Published:** 2026-08-14

**Authors:** Melanie A. Gunawardene, Prashanthan Sanders

**Affiliations:** 1 Centre for Heart Rhythm Disorders, Adelaide University and Royal Adelaide Hospital, Adelaide, SA, Australia; 2 CCB, MVZ CCB Frankfurt und Main Taunus GbR, Markus Hospital, Frankfurt am Main, Germany; 3 Department of Cardiology, University Hospital Giessen, Giessen, Germany

## What a time to be an electrophysiologist

There has never been a more exciting time to pursue a career in cardiac electrophysiology. Pulsed field ablation is redefining how we approach cardiac ablation, 3-dimensional mapping systems are becoming increasingly sophisticated, procedural imaging is transforming how we visualize anatomy in real time, and artificial intelligence is beginning to reshape our understanding of arrhythmia mechanisms. For fellows and early-career electrophysiologists, this convergence of technologies is not just a clinical reality to adapt to. It is an invitation. A career in electrophysiology today extends far beyond the laboratory, opening doors to research, industry, startups, and innovation. The key is not to wait until you feel ready. In a nascent field, nobody is truly ready. When a technology is new, everyone starts from the same place, and that is precisely where opportunity lives.

## Choose your frontier

Every new technology arrives with more questions than answers. Efficacy, efficiency, and safety all demand rigorous investigation, and for the early-career electrophysiologist, this abundance of unanswered questions represents a genuine academic opportunity. The key is selectivity: identifying a specific subtopic and pursuing it with depth and consistency. Gaining this expertise helps build a reputation. Starting with your own institutional data is a valuable first step. Even a single-center experience, rigorously described, adds meaningfully to the evidence base. From there, collaboration opens new doors, as multicenter datasets answer questions that no single center can address alone. Those who remain actively engaged in advancing the efficacy, safety, and mechanistic understanding of a technology will find there is always a place for their contribution and always an audience eager to engage.

## Be in the room

In the summer of 1790, Alexander Hamilton, Thomas Jefferson, and James Madison met privately over dinner to strike one of the most consequential political deals in American history. Excluded from that dinner, Aaron Burr could only watch from the outside, a frustration later immortalized by Lin-Manuel Miranda in the musical Hamilton: “I want to be in the room where it happens.” The lesson for early-career electrophysiologists is the same: the decisions and discoveries that shape a field rarely emerge from publications alone. They happen in laboratories, at congresses, and in the conversations between sessions. Time spent in the laboratory, observing and participating across the full spectrum of cases, builds a clinical foundation that no publication can replace. Equally important is engaging with the technology at its roots, visiting congresses such as HRX where industry engineers, scientists and clinicians converge, and building relationships with research and development teams. In the early phase of any emerging technology, opinion shapes practice before evidence exists. This makes 2 things essential: first, being present in the conversations where the field is being shaped, and second, taking the questions raised in those rooms back home to generate the evidence that is not yet there but urgently needed.^1^^,^^2^ The best research questions emerge from the discussions, debates and unresolved challenges that fill the rooms where the field converges.

## Good news is not the only news

One of the most liberating lessons for early investigators is that impactful science does not require positive findings. A safety signal, an unexpected observation, a finding that challenges prevailing assumptions: these contributions are equally valuable, provided they are reported honestly and interpreted with rigor. During the early clinical experience with pulsed field ablation at one of the first centers to adopt the technology, an unexpected case of coronary spasm was observed and documented, the first such report in the context of PFA.^3^ Rather than a setback, this observation became the foundation for a dedicated research program encompassing systematic data collection, collaborative publications and an ongoing investigation into the mechanistic basis of this phenomenon.^4^^,^^5^ The field advances not only through confirmatory evidence, but through the courage to report what is actually seen. For young electrophysiologists, the lesson is simple: the clinic is not just where we apply knowledge; it is where we find the questions that matter most.

## Your moment is now

The electrophysiologists who shape the evidence base for emerging technologies share one defining characteristic: they chose to engage with what was new and uncertain. Every generation has its frontier. The question is not whether you were there at the very beginning. It is whether you are willing to step into the room today and write the first chapter of your own story.

## Funding Sources

This research did not receive any specific grant from funding agencies in the public, commercial, or not-for-profit sectors.

## Disclosures

M.A.G. receives speaker’s honoraria, educational grants, fellowship, consultation fees from Boston Scientific/FARAPULSE Inc., Medtronic, Biosense Webster/J&J Medtech, Abbott, Biotronik, Zoll, and Bristol Myers Squibb; and is supported by a postgraduate scholarship from the University of Adelaide. P.S. reports that the University of Adelaide has received research funding, on his behalf, from Medtronic, Abbott Medical, Boston Scientific, and Becton-Dickenson; and reports serving on the advisory board of Medtronic, Abbott Medical, Boston Scientific, CathRx, and Pacemate.
